# Ensemble-based multi-tissue classification approach of colorectal cancer histology images using a novel hybrid deep learning framework

**DOI:** 10.1038/s41598-023-35431-x

**Published:** 2023-05-31

**Authors:** Masoud Khazaee Fadafen, Khosro Rezaee

**Affiliations:** 1 Department of Electrical Engineering, Technical and Vocational University (TVU), Tehran, Iran; 2 Department of Biomedical Engineering, Meybod University, Meybod, Iran

**Keywords:** Engineering, Biomedical engineering

## Abstract

Colorectal cancer (CRC) is the second leading cause of cancer death in the world, so digital pathology is essential for assessing prognosis. Due to the increasing resolution and quantity of whole slide images (WSIs), as well as the lack of annotated information, previous methodologies cannot be generalized as effective decision-making systems. Since deep learning (DL) methods can handle large-scale applications, they can provide a viable alternative to histopathology image (HI) analysis. DL architectures, however, may not be sufficient to classify CRC tissues based on anatomical histopathology data. A dilated ResNet (dResNet) structure and attention module are used to generate deep feature maps in order to classify multiple tissues in HIs. In addition, neighborhood component analysis (NCA) overcomes the constraint of computational complexity. Data is fed into a deep support vector machine (SVM) based on an ensemble learning algorithm called DeepSVM after the features have been selected. CRC-5000 and NCT-CRC-HE-100 K datasets were analyzed to validate and test the hybrid procedure. We demonstrate that the hybrid model achieves 98.75% and 99.76% accuracy on CRC datasets. The results showed that only pathologists' labels could successfully classify unseen WSIs. Furthermore, the hybrid deep learning method outperforms state-of-the-art approaches in terms of computational efficiency and time. Using the proposed mechanism for tissue analysis, it will be possible to correctly predict CRC based on accurate pathology image classification.

## Introduction

Globally, colorectal cancer (CRC) accounts for around 10% of all cancer deaths^1^. This disease caused approximately 1.09 million new cases and 551,000 deaths in 2018. In 2030, the World Health Organization estimates that 75 million people will suffer from CRC, 17 million will die, and 27 million new cases will be diagnosed^2^. The clinical outcomes of patients with resectable cancer can vary widely, however. In addition, there is evidence that survival is associated with tumor symptoms, such as smoking, diabetes, obesity, nutritional deficiencies, and diabetes mellitus^3^. 60–80% of CRC recurrences occur within the first 2 years after resection, and 95% within the first 4 years^4^. Fine-needle aspiration (FNA) or needle biopsy (NB) are the most commonly used methods for detecting colon tumors^5,6^. Through a microscope, samples of cells or tissues are viewed on unique glass slides. As a result, histopathology images (HI) are well-established and reliable for determining CRC. HI analysis is useful for the clinical assessment of CRC^7,8^. To accurately analyze and treat CRC, it is necessary to describe tumor regions, assess aggressiveness, and classify carcinoma prototypes from full slide images^9^. CRC is diagnosed by examining tissue samples under a microscope, staging and grading them with a microscope.

In HI analysis, which can take a considerable amount of time, pathologists' expertise and talents are crucial. The HI analysis process is also associated with damaging variables such as exhaustion and an inability to focus on problematic skills. Automated models have been improved to enhance the efficiency and precision of CRC diagnosis. Efforts have been made to improve their decision-making outcomes^10^. Computer-assisted diagnosis (CAD) algorithms can be expanded through the development of image processing algorithms and machine learning approaches. Additionally, it helps to facilitate decisions and reduces the amount of time spent interpreting and evaluating HI. Automatic detection systems make it possible to detect CRC early, which can lead to proper treatment. The primary challenge is, however, determining how to extract meaningful features from HI obtained from colon tissue. While hand-crafted feature extraction methods assist in defining tissues' states, they are unreliable in terms of obtaining discriminating feature vectors that aid in classification.

Deep learning (DL) models have recently shown significant improvements in decision-making in CAD systems and medical applications^11,12^. DL networks cannot function without large amounts of data, high training costs caused by complex data patterns, and the absence of standard hypotheses for selecting an appropriate DL structure^13,14^. DTL is a machine learning approach in which a pattern developed for one task is used as a basis for building another^15,16^. As a starting point for a secondary assignment, DTL techniques use a transfer learning architecture^17^.

Several previous works have employed hand-crafted features to address low classification rates, computational complexity, and low-quality HI. A malignant tumor's texture, morphology, and statistical characteristics are described in Ref.^18^. Accordingly, their methodology relies on feature embedding and unsupervised clustering^19^. Although traditional ML procedures require an expert to extract features, DL structures can be extracted without expert knowledge^20,21^.

CRC cancers have rarely been classified using DTL-based analysis of histopathology images in previous publications. CRC research has used DL techniques based on HI analysis, however.

Based on tissue interpretation, automatic CRC detection applications classify HI into benign and malignant types. Due to its inherent complexity, HI is a major concern in these systems. Analysis and diagnosis of HI have occupied a significant amount of time in the investigation. For the automatic classification of malignant and benign tissues, automated computer applications are used as CAD systems.

A pathologist's biggest challenge is identifying the cancer's grade. There are several classes of CRC that have been considered in quantitative research. Identification of a tissue type aids the treatment process. This challenge is plagued by overfitting, which results in a lack of accuracy. Due to the high similarity of textures between the images, an automated learning method is required. By using DL strategies, absolute errors can be reduced. For CRC classification and automation applications, deep learning requires many images.

An improved DTL model for the classification of CRC is presented in the present work. HI can be classified with satisfactory accuracy for a large number of colon tissue classes and a modest number of training images using the improved residual neural network (ResNet). The effectiveness of ResNet's architecture as a model for feature extraction has previously been evaluated by comparing it with other similar methods under the same conditions. ResNet reduces the number of layers and improves the quality of feature maps compared to prior designs such as DenseNet and other CNNs. However, a number of issues and shortcomings have been identified.

1. Conventional image processing makes it difficult to identify and interpret HI and distinguish features of specific diseases.
2. Different disease stages require systematic assessment of the attributes of disease patterns using various images.
3. Handcrafted features determine the effectiveness of machine learning approaches. For this reason, feature extraction must be automated in order to select and learn the most appropriate set of features.
4. There are deep learning models that use well-known architectures, such as transfer learning. Since it can classify millions of images, such models can be implemented quickly, provided there is a trade-off between computational load and accuracy.
5. The deep learning network should be trained on a large number of images to ensure that the features are more generalized.

A major obstacle to HI is the lack of labeled images as well as the variability of images due to staining. HI is more about textures than well-defined objects, making obtaining a histopathological image challenging. This work used a modified deep model and ensemble learning technique using SVMs with RBF kernels. The study aimed to fill research gaps in CRC classification in HI. Among the study's notable contributions are:

- The research presented in this area presents a distinctive and innovative DL architecture. The first objective is to improve feature classification using hue, saturation, and value (HSV) color space. When images of histopathological lesions are converted from RGB to HSV color space, colors appear more accurate. Their light intensity is also more homogeneous and balanced. The second stage uses TL to acquire and enhance performance through the acquisition of different feature maps.
- To our knowledge, this is the first time that a hybrid structure based on a modified deep TL network and ensemble learning has been used to detect CRC in a large number of HIs.
- Eight classes of CRC are examined through HI analysis. Moreover, the proposed method can significantly reduce obstacles such as uncertainty and generalizability. It is expected that this method will be robust to different conditions, such as inhomogeneity or the absence of high-quality HI.
- A variety of methodologies were used to train and compare the algorithm. Through hold-out cross-validation, 100,000 images from two HI datasets were used to train and validate the architecture.

The structure of our research is as follows. Section “Related work” discusses related research. The introduced feature extraction approach based on the modified DTL structure, feature selection, and ensemble learning is described in “The proposed approach”. The experimental outcomes obtained with the suggested method for HI analysis are explained in “Experimental results”. The study concludes with a summary of the major themes discussed in “Conclusion”.

## Related work

Medical image processing is only one example of how ML's related algorithms and approaches have become more ubiquitous due to their efficacy. Moreover, a variety of methods are available in ML to reduce the dimensions of features, including feature selection, feature projection, and feature reduction^22^. The implementation of DL to diagnose colon cancer has received more attention in most previous histopathological imaging studies because the disease has a high mortality rate^23–27^.

Contrary to conventional strategies, which extract general shapes or textures from HI, DL-based methods learn a discriminative description directly from input HI. By using CNN structures and active contour segmentation, Haj-Hassan et al.^28^ have developed a method for classifying CRC tissues from multi-spectral HI. They predicted three tissues types associated with CRC grades, including benign hyperplasia (BH), intraepithelial neoplasia (IN), and carcinoma (Ca), and reached an accuracy of 99.17% for segmented HI regions.

Study Iizuka et al.^29^ applied trained recurrent neural networks (RNNs) and CNN structures to whole-slide images (WSIs) to diagnose stomach and colon cancers. Based on three types of tissues: adenoma, adenocarcinoma, and non-neoplastic, they classified WSI images with 96–99% accuracy.

Masud et al.^30^ introduced a classification system for discriminating between five types of lung and colon tissues, including two benign and three malignant, according to how their HI is interpreted. Based on their research, the developed framework has a maximum accuracy of 96.33% for recognizing cancerous tissues in HI.

Using HI analysis and features interpretation for lymph node metastasis (LNM) in CRC, Kwak et al.^31^ presented an accurate CAD system. To identify CRC tissue using multiple data, the researchers developed a DL model based on CNN structure.

Rezaei et al.^32^ introduced a scheme based on the LinkNet structure for gland segmentation, and they examined the impact of applying different loss functions. The Warwick-Qu dataset, which comprises two data sets, demonstrated that their strategy is comparable to similar approaches.

Sirinukunwattana et al.^7^ have extended a DL architecture for detecting and classifying nuclei into four types (miscellaneous, inflammatory, epithelial, and fibroblastic). Korbar et al.^33^ have also designed a CAD system to help pathologists depict colon polyps.

Xu et al.^34^ presented an algorithm that reduced heavy feature design by applying CNNs to a deep multi-channel framework and could meet various needs by changing channels. Comparing their approach to the approaches reported in the 2015 MICCAI Gland Segmentation Challenge and to other segmentation methods, they evaluated the results according to the same criteria to demonstrate its superiority.

The HI of colon regions was used as a basis for the segmentation of glandular structures by Manivannan et al.^35^. The authors employed a structured learning framework that illustrates the spatial configuration of class labels and captures structural information that is often missed by sliding window methods. To learn the support vector machine classifier, they obtained samples of label structures through clustering. In the end, they combined hand-crafted, multi-scale image features with features estimated by a DL trained to map outcomes to segmentation projections. The resulting system was tested using the GlaS dataset.

Based on the Faster-RCNN-based convolution neural network structure, Ho et al.^36^ incorporated a deep learning model constructed on spinal segmentation of the ResNet-101 feature that excluded gland segmentation. With a sensitivity of 97.4%, the validation group achieved an AUC of 0.917 in recognizing high-risk features of malignancy and dysplasia.

In the first stage of colorectal histopathology image categorization, Chen et al.^37^ employed CNNs and multi-channel attention mechanism models to extract information for classification. They added misclassified images to the training set repeatedly in the second stage, improving the model's performance. They achieved classification accuracy of 98.98% on their own dataset and 99.77% on the HE-NCT-CRC-100K dataset, respectively.

Based on the CNN structure, Wang et al.^38^ suggested a new patch aggregation technique for diagnosing CRC clinics by utilizing poorly labeled diseased slide images patches. Their technique was trained and validated on a large number of HIs. A kappa of 0.896 was their average. Area under curve (AUC) was much higher than that of pathologists (0.988 vs. 0.970) and outperformed other comparable approaches for CRC diagnosis.

Utilizing histopathological pictures in a variety of configurations, Riasatian et al.^39^ introduced KimiaNet, a DenseNet-based network composed of four dense blocks. In the Cancer Genome Atlas (TCGA) library, there are 7126 full slide images of formalin-fixed paraffin-embedded human pathology samples generated from 240,000 image patches taken at a magnification of 20. Three public datasets were used to evaluate KimiaNet's search and classification performance: images of colorectal cancer, endometrial cancer, and the TCGA.

To diagnose colon cancer from visual data, Yildirim et al.^40^ developed a CNN-based, MA ColonNET system. In order to categorize these cases, they used the 45-layer model of MA ColonNET. This structure has an accuracy rate of 99.75%.

The categorization of CRC was carried out using ML approaches by Alqudah et al.^41^. They use three distinct color spaces to extract features from a 3D Gray Level Co-occurrence Matrix (GLCM). With a testing dataset of 1496 images and a training dataset of 3504 images, 3D GLCM matrices of the pictures were produced and analyzed. According to their study, the best ML model achieved testing and training scores of 97% when using Quadratic Discriminant Analysis (QDA).

Using the CNN architectures InceptionV3, DenseNet201, MobileNetV2, ResNet152, ResNet101, VGG19, and VGG16 for classification, Ref.^42^ presents a method for predicting the CRC. Using a 10,000-image dataset, they divided it into 3200 images: 7200 for training, 1800 for validation, and 1000 for testing. VGG19, ResNet152, and ResNet101 are the three architectures that successfully classify and identify both types of CRC.

Based on two publicly available datasets, Kumar et al.^43^ constructed a lightweight, less complicated CNN for categorizing multiclass colorectal tissue HIs. HIs are fed to pre-trained models (VGG16, Xception, DenseNet121, and InceptionResNetV2) and the proposed method. Training the created network took less time than training other TL methods. The presented network achieved 93.50% accuracy on the colorectal histology dataset and 96.26% accuracy on the NCT-CRC-HE-100K dataset.

An overview of the literature reviewed can be found in Table 1.

**Table 1:** A summary of the literature review is shown in this table.

Authors	Year	Method	Dataset	No. of classes	Performance
Sakr et al.^23^	2022	CNN	LC25000	2	Acc: 99.50%
Wilm et al.^24^	2022	CNN	Two HIs	7	Acc: 93.8–95.7%
Moyes et al.^25^	2023	Multi-channel auto-encoder	Synthetic dataset	9	F-score: 0.620738
Gavade et al.^26^	2023	ResNet-50	Kaggle	2	Acc: 98.9%
Li et al.^27^	2023	Embedded fusion mutual learning	LC25000	2	Acc: 98.96% AUC: 0.9973
Haj-Hassan et al.^28^	2017	CNN	CHU Nancy Brabois Hospital	3	Acc: 99.17%
Iizuka et al.^29^	2020	CNN and RNN	Hiroshima University Hospital	3	AUC: 0.97–0.99
Masud et al.^30^	2021	CNN	LC25000	5	Acc: 96.33%
Kwak et al.^31^	2021	CNN	Portal GDC	7	–
Chen et al.^37^	2022	Multi-channel attention	HE-NCT-CRC-100K	9	Acc: 99.78%
Yildirim et al.^40^	2022	CNN	Kaggle	2	Acc: 99.75%
Alqudah et al.^41^	2022	QDA	Kaggle	8	Acc: 97.30%
Kumar et al.^43^	2023	CNN	NCT-CRC-HE-100K	9	Acc: 99.21%

In comparison to prostate and breast and tissue, CNN in colonic histopathology is still in its infancy. With our hybrid model with modified DTL structure, we can address such concerns as overfitting, inadequate learning, uncertainty, etc. Instead of previous research, which relied solely on established datasets or binary classes (tumor or not tumor), we constructed our own segmentation model independent of existing datasets and trained it and tested it on a wide array of training data. ML can detect epithelial tumors despite non-neoplastic background when applied to colonic biopsy WSI using a highly functioning CNN. In the specialized field of colonic histology, this further emphasizes DL’s importance.

## The proposed approach

**Figure 1:**
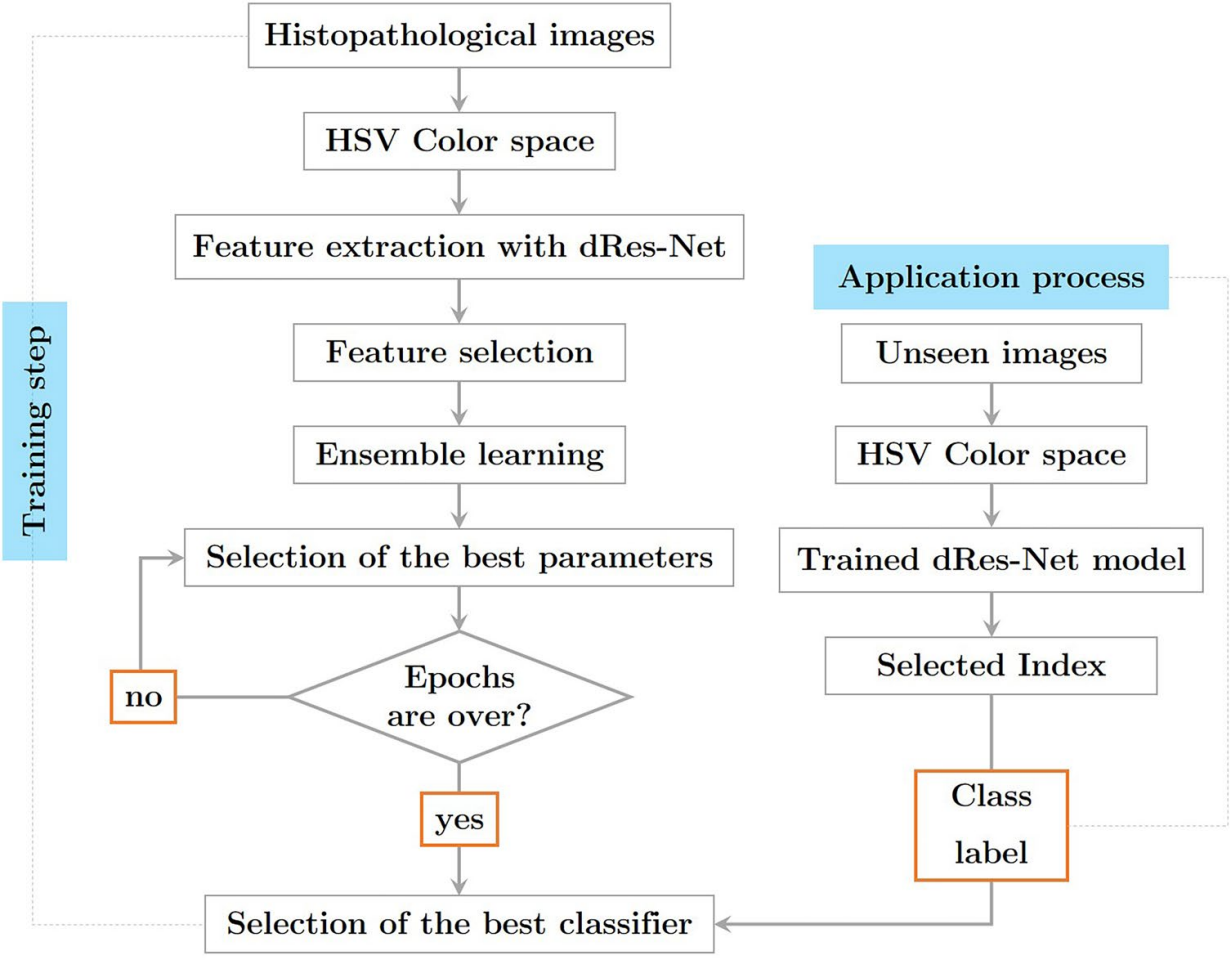
The steps of the introduced method are illustrated in 2 sections: training and testing.

Figure 1 reveals the general structure of the introduced procedure for detecting CRC in HI. Each section of the method is described below.

### HSV space

**Figure 2:**
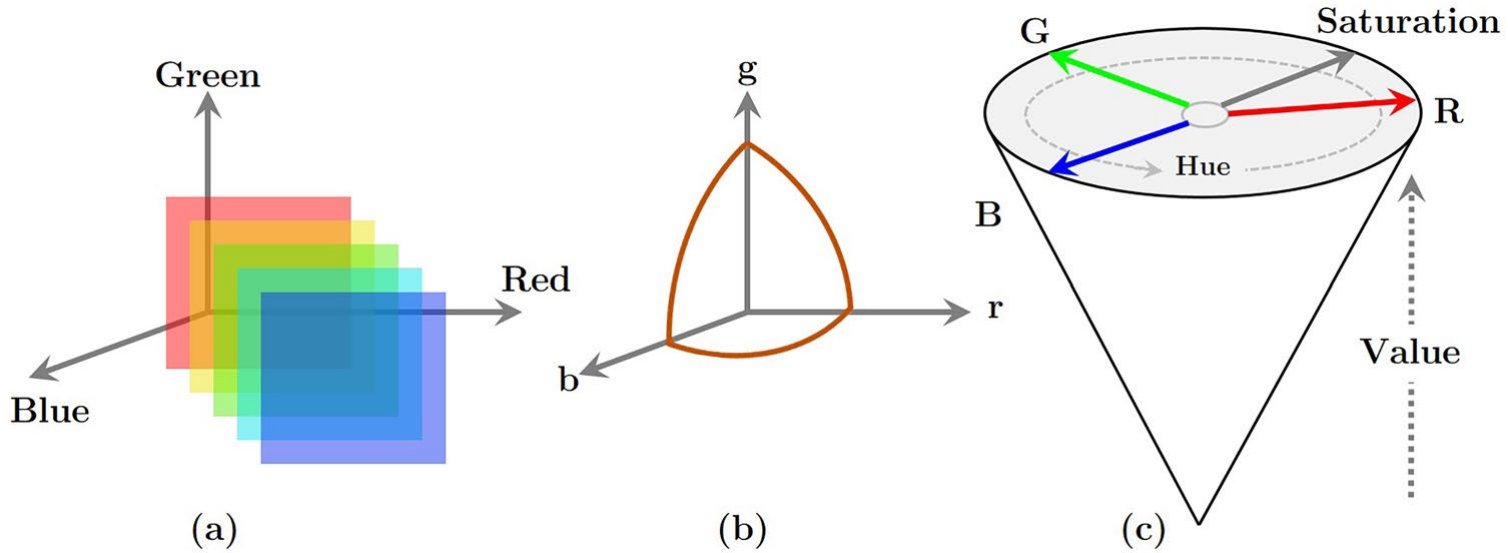
Schematic representation of the stages of converting a (**a**) RGB image to (**c**) HSV space. The plot (**b**) is rgb coordinates to convert RGB to HSV space.

To create homogeneity with natural light, a HSV (Hue, Saturation, and Lightness) display is needed for the HI processing step. There is a tendency to use the terms "HSV" and "white light" interchangeably since the strongest hue of HSV resembles white light (e.g., a bright white light shining on a red surface). In low light, objects that appear redder and brighter in high light appear darker and brighter. To ensure that no light is lost during the HI analysis, a single point source must be obtained. A pre-processed RGB image is used to keep the brightness constant in the HSV converter. The procedure of converting an RGB image to an HSV image is illustrated in Fig. 2.

### Feature extraction

Gradient dissipation or explosion is the first problem with increasing network depth. Gradients propagating through a network become unstable as more layers are added, becoming either very large or very small. Dissipation is a common occurrence throughout time. We can use ReLU activation, batch normalization, and a variety of additional strategies to prevent gradient dissipation. As efforts have been made to minimize gradient dissipation, the problem arises when the depth of the network increases. With more layers in the network, the network may be able to extract more difficult feature patterns, meaning a deeper model should provide better results. However, with more layers in the network, the error of classification may become greater. Moreover, it is not related to excessive fitting, since the accuracy of the training set is also decreased. Fortunately, the residual network in ResNet structures solves the problem, and as a result, the network depth increases several times.

Based on 3 × 3 VGG full-layer architecture, ResNet is built. There are two 3 × 3 convolutional layers with an equal number of output channels in the residual block. A batch layer, a ReLU, and a normalized convolutional layer were added after each convolutional layer. In addition, we repeat these two convolved computations, including the input shortly before the final ReLU activation function. Two convolutional layers are combined into a single type of input. This channel count will need to be increased. There is a need to add an additional 1 × 1 convolutional layer to calibrate the input. The input is added to the output before the non-linear ReLU is performed. Only the channels will be altered before the 1 × 1 convolutional layer is introduced. Although the ResNet transfer learning structure is very effective at recognizing objects of different sizes, there are still challenges in recognizing objects of different sizes in images.

**Figure 3:**
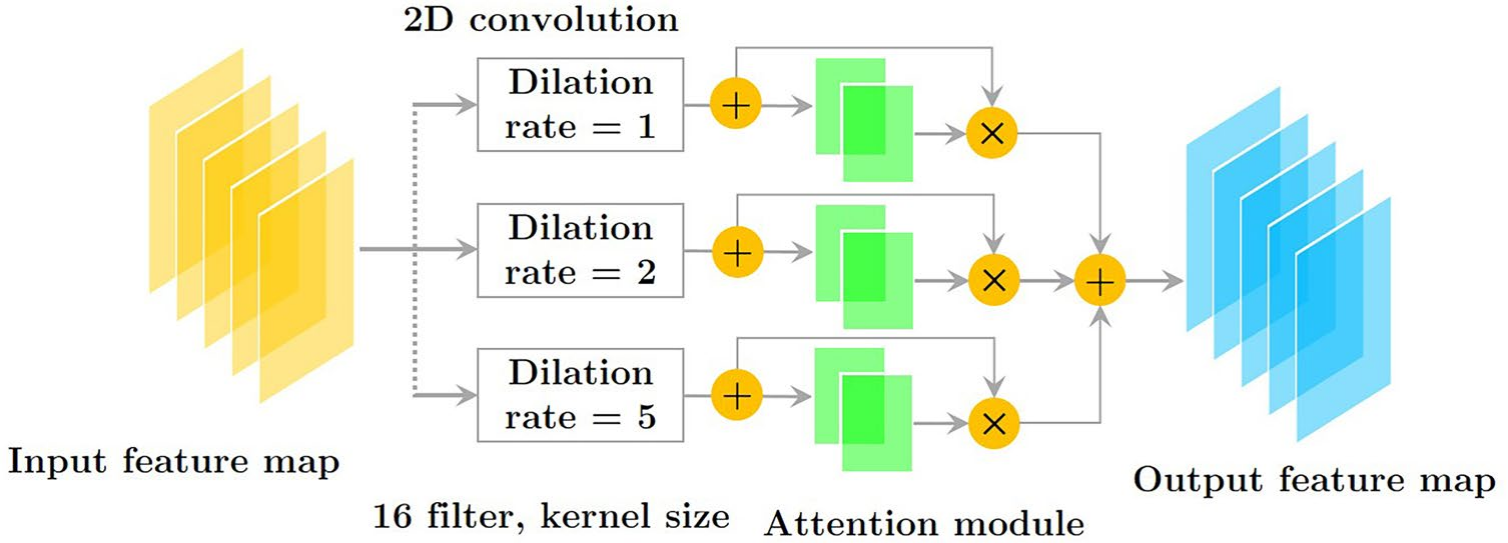
The structure of dilated convolution block.

**Figure 4:**
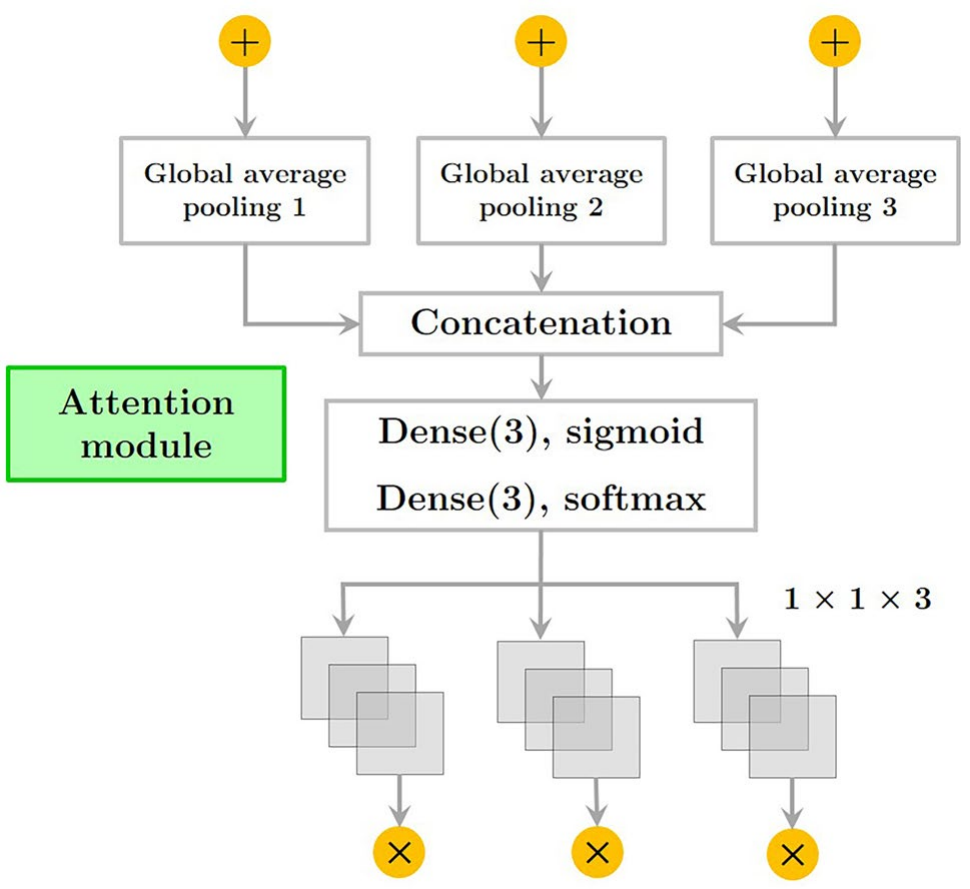
The architecture of attention module.

Dilated convolution has gained popularity in recent years due to its ability to enhance the kernel's receptive field without adding extra parameters. The convolution kernel is what separates dilated convolution from standard convolution. In the dilated convolution kernel, only a subset of the locations corresponds to learning parameters; the remainder are left blank. Figures 3 and 4 show the architecture of a dilated version of convolution block as well as the display of a dilated convolution (DiConv). The total resolution of the image is less than 1% occupied by a small object. The coarsest, deepest layer of the ResNet architecture fails horribly at expressing the minute properties of tiny objects. Dilated ResNet (dResNet) generates predictions about the target at three different scales (Dilation rate = 1, Dilation rate = 2, Dilation rate = 3). The dResNets method requires three anchor boxes per grid cell at all resolutions. We use residual blocks carrying high-detail information in HIs in order to differentiate objects of different sizes, enhance spatial resolution by using dilated convolution and upsampling, and concatenate them together.

ResNet's well-known shortcoming is addressed by utilizing small items in a multi-layered, high-dimensional environment. The sampled layers combine with the previous layers to preserve the architecture's fine-grained characteristics and to detect objects of varying sizes. A dilated convolution formula is shown in Eq. (1):

1 $$y(u, \, v) \, = \sum\limits_{m} {\sum\limits_{n} {s(u + k \cdot r,v + l \cdot r) \cdot h(k,l)} }$$

Dilation rate is specified by *r*. This shows that the receptive area of DiConv is greater than standard convolution with the same number of parameters. Additionally, DiConv allows for more efficient aggregation of global data. The parallel paths were convolutionized at 1, 2, and 5 dilated convolutions per second. An attention-based method was used to improve feature fusion. In the first step, each parallel dilated convolution feature map is pooled globally. By activating all of the linked layers, their weights can be established. In the end, the dilated convolution block is considered the output of the weighted fused feature map. The general process is summarized as follows:

2 $$A_{i} = GlAtPo(D_{i} )$$

by dilating the convolution with different dilation rates, we get *D*_*i*_ as the feature map. Furthermore, global average pooling operation are also known as *GlAtPo*.

3 $$\eta_{i} = \sigma_{1} (Dense(A_{i} ))$$

where, layers with dense connections are fully interconnected. The SoftMax function and sigmoid operator and the are denoted by σ_1_ and σ_2_.

4 $$\mu_{i} = \sigma_{2} (Dense(\eta_{i} ))$$

Finally, *Output* is the final outcome of the DiConv block, where *µ*_*i*_ is the weight of the feature map *D*_*i*_.

5 $$Output = \sum\limits_{i = 1}^{3} {\mu_{i} .D_{i} }$$

### Feature selection

As a result of deep structure feature extraction, we further select the features with the least computational complexity. We can compute the weights of neighborhood features by using distance measures. Neighborhood component analysis (NCA)^44^ can be employed to reduce the size of the requisite feature vector by combining "non-parametric and supervised" techniques.

This method allows us to decrease the amount of feature vectors obtained from HI. The NCA is able to compute feature rank since each feature is assigned a positive weight. The NCA is employed to estimate feature weights when applying a desired feature reduction model. The features are divided up into overlapping blocks before being reduced. Therefore, we can conclude that k is a collection of smaller vectors.

### Ensemble learning

The ensemble method is used in ML and statistics to improve prediction performance over individual learning algorithms^45^. Ensemble models have two major, interconnected advantages over single models: they provide better performance and predictability than individual components. Moreover, in addition to reducing variance in predictions, an ensemble increases robustness. Contrary to the limitless statistical ensembles, ML ensembles contain a finite number of distinct models, but typically allow for a more flexible structure among them. In the D-dimensional space, each input in a training dataset is a point, implying that the training dataset contains *D* components. The hyperplane with the largest margin of error is generated after mapping the training data onto a higher-dimensional feature space. This outcomes in a nonlinear decision boundary in the input space. When determining the separating hyperplane, it is possible to use kernel functions such as polynomials, spline, and radial basis functions (RBFs). On the basis of the linearity of the dot product, we can construct the decision function as follows:

6 $$f({\mathbf{x}}) = {\text{sgn}} \left[ {\sum\limits_{j = 1}^{l} {y_{j} \alpha_{j} \cdot ({\mathbf{x}} \cdot {\mathbf{x}}_{j} ) + b} } \right]$$

A high-dimensional feature space can be derived from a non-linear transformation of an input vector set (x_1_, …, x_l_). Whenever deciding what to do, one must:

7 $$f({\mathbf{x}}) = {\text{sgn}} \left[ {\sum\limits_{j = 1}^{l} {y_{j} \alpha_{j} \cdot } K({\mathbf{x}},{\mathbf{x}}_{j} ) + b} \right]$$

Besides, support vectors can make use of kernel RBF as explained in Eq. (8):

8 $$K({\mathbf{x}},{\mathbf{x}}_{j} ) = \exp ( - \left\| {{\mathbf{x}} - {\mathbf{x}}_{j} } \right\|^{2} \times (c)^{ - 1}$$

**Figure 5:**
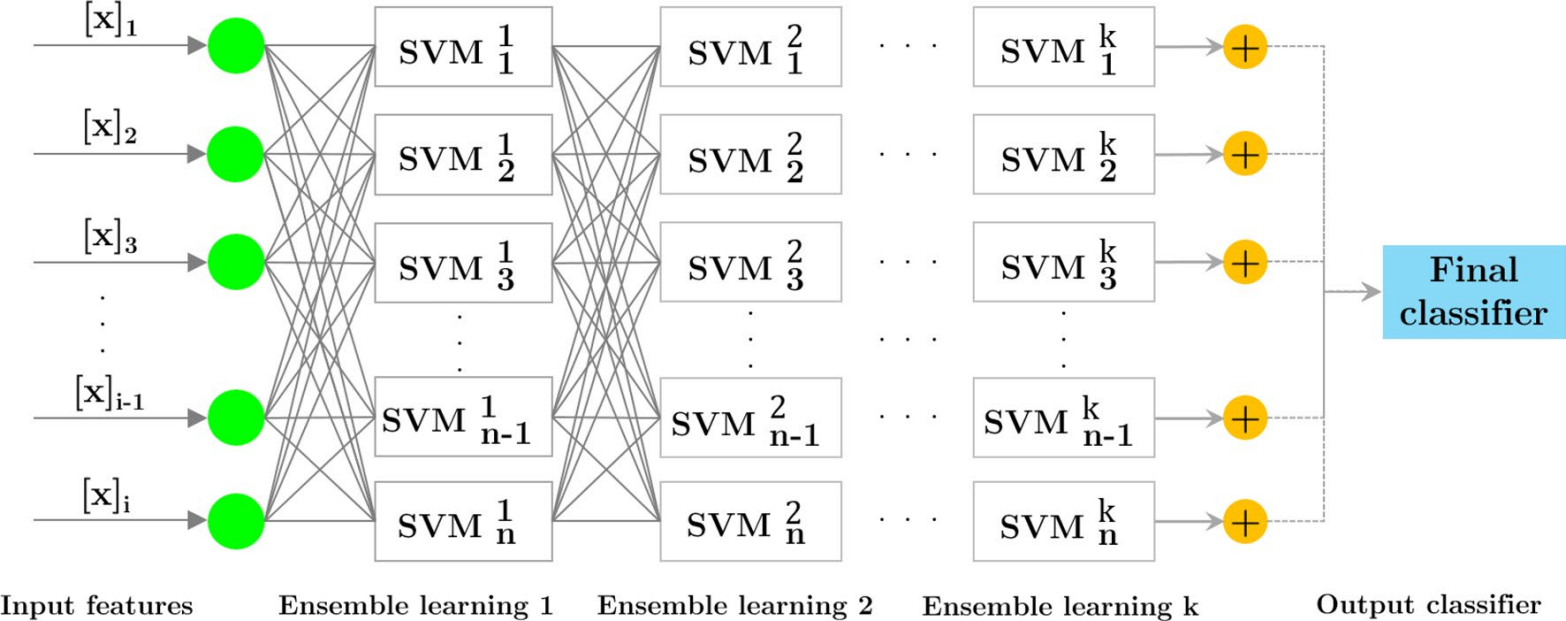
An ensemble model based on the pool of SVMs is shown in this figure.

A computed RBF kernel in Eq. (8) contains fewer hyper-parameters, more variables, and a simpler mathematical structure than other kernels. Due to these characteristics, it has been widely accepted. When the classification step is performed, we use the multi-SVM with a RBF-based learning pool of the ensemble learning technique. Figure 5 illustrates the extended ensemble learning framework. DeepSVM is based on the ensemble structure, and it utilizes the conventional multi-SVM structure and RBF kernel from^46^.

The layers of this structure are each taught using supervised learning. A classifier uses labels to generate brand-new training data at every level in the classification process. It has been demonstrated that this approach provides effective parameter values for the classification procedure.

Figure 5 shows a multilayer architecture with an input layer, hidden layers, and an output layer. After that, *n* SVMs are employed to translate new features into the next layer. A model's performance directly depends on selecting the appropriate classifier for each layer and quantifying the value of each feature.

Algorithm 1 illustrates an ensemble learning structure based on multi-SVM classifiers. Using SVM, the hidden layer's feature vector is trained before constructing the decision function. Due to the high discriminant nature of dResNet's features, the NCA's feature selection is constrained, and as a result, the DeepSVM structure is not overburdened with computational complexity. Ensemble-based classification can be implemented in two ways: one uses reduced-dimensional feature vectors as training data, while the other uses the final classifier.
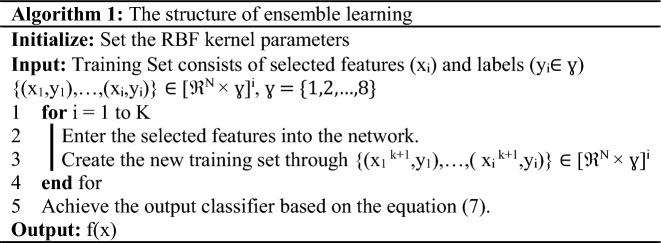


## Experimental results

### Dataset

**Figure 6:**
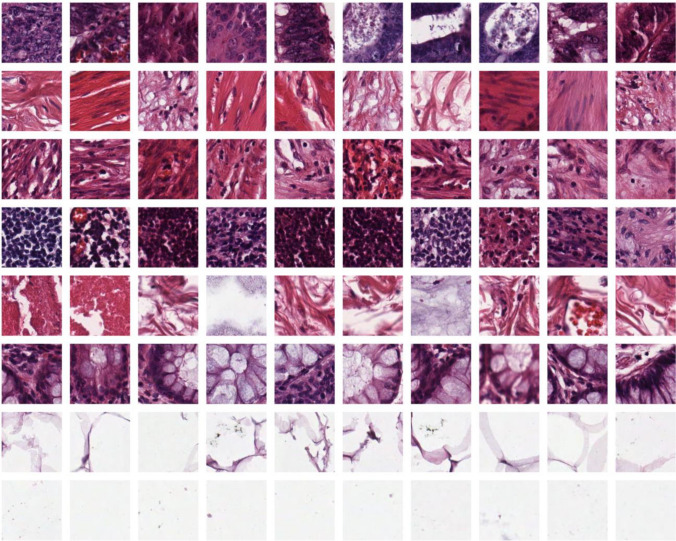
Single rows containing examples from a single class are used to illustrate sample images from a data set. The HIs from left to right are: (1) tumor epithelium, (2) simple stroma, (3) complex stroma, (4) immune cells, (5) mucosal remnants, (6) mucosal glands, (7) adipose tissue, and (8) background^47^.

There is a public dataset available from the University Medical Center Mannheim (Germany)^47^. Digitalized colon cancer tissue slides contain samples of tissue from low- and high-grade primary tumors. The Fig. 6 depicts eight different textures found in tumour samples: (1) the epithelium of the cancer (TUMOR), (2) the cells of the stroma (STROMA), (3) the tissue of the stroma (COMPLEX), (4) the immune cells, (5) the mucus and debris (DEBRIS), (6) the glandular mucus (MUCOSA), (7) the adipose tissue (ADIPOSE), and (8) the background (BACK). There are 5000 image tiles in the dataset with dimensions of 150 × 150 pixels and 74 µm × 74 µm. They are 20 times clearer and contain more formalin and other histopathological markers, so the pathologist can easily diagnose them. The labels for each image have also been reviewed by the Institute of Mannheim University of Medical Sciences in Germany. As well as the train, test, and validation images, Table 2 shows the details of the first dataset (Kather texture 2016).

**Table 2:** Data setting for Kather texture 2016 for training, validation, and testing.

Class	Image type	No. train images	No. validation images	No. test images
1	TUMOR	375	125	125
2	STROMA	375	125	125
3	COMPLEX	375	125	125
4	MUCOSA	375	125	125
5	DEBRIS	375	125	125
6	MUCOSA	375	125	125
7	ADIPOSE	375	125	125
8	BACK	375	125	125
Total	All	3000	1000	1000

Another publicly available CRC dataset, NCT-CRC-HE-100K^48^, contains 100,000 patch-level images of nine separate tissue categories, each with an aspect ratio of 224 × 224 pixels and an average pixel size of 0.5 μm. In total, nine distinct tissue types can be analyzed, including the nine different categories of tissue are colorectal adenocarcinoma epithelium (TUM), cancer-associated stroma (STR), normal colon mucosa (NORM), smooth muscle (MUS), mucus (MUC), lymphocytes (LYM), debris (DEB), background (BACK), and adipose (ADI). In addition to the train, test, and validation images, Table 3 shows the details of the second dataset.

**Table 3:** Data setting for NCT-CRC-HE-100K for training, validation, and testing.

Class	Image type	No. train images	No. validation images	No. test images
1	TUM	8591	2863	2863
2	STR	6268	2089	2089
3	NORM	5258	1753	1753
4	MUS	8122	2707	2707
5	MUC	5338	1779	1752
6	LYM	6935	2311	2311
7	DEB	6908	2302	2302
8	BACK	6340	2113	2113
9	ADI	6245	2081	2081
Total	All	60,005	19,998	19,997

### Setting

This algorithm is implemented on a system with a Core i-7 processor and 8 GB of RAM. The version of MATLAB used is the 2020b version, which has online plugins and also has the latest updated version of this software's toolbox.

There is no separate electronic graphics processing unit (GPU) hardware board in the system used. By dividing the data in a 60–20–20 ratio, we can create training, testing, and validation sets. As a complement to the main program, SPSS was also used. Afterward, the RBF kernel is configured in DeepSVM to achieve the best possible results. It employed the learning rate (*µ*) of 0.002 and a range of epochs between 500 and 2000 in its initial model. By utilizing an ensemble learning system that employs parameter adjustment and early stopping in order to determine the optimal training iteration size, overfitting can be avoided.

### Evaluations

We compared ResNet family-based learning strategies with dResNet architecture as a first step towards identifying possible improvements in the classification of CRCs based on HI. In both datasets, dResNet-101 is implemented to ensure that the feature extraction stage performs as efficiently as possible, as demonstrated in Tables 4 and 5. According to Table 2, each of the classes (i. e., Tumour epithelium = 1, Stroma (simple) = 2, Stroma (complex) = 3, Immune cell conglomerates = 4, Debris and mucus = 5, Glands = 6, Adipose = 7, Background = 8) could be recognized in the first dataset by multi-class categorization. While ResNet deep transfer learning algorithms were used for feature extraction, the rest of the algorithm remained largely unchanged. As an example, the best selected classifier resulted from ensemble learning, and NCA-based features with the same number of features were utilized to achieve fair comparison. As DeepSVM aids the classification procedure, ensemble-optimized support vector machines are effective. Similar feature extraction methods are also employed by ResNet-164, ResNet-152, and ResNet-101.

**Table 4:** In this table, the outcomes of CRC classification utilizing HI for the first dataset with eight classes are presented. The bolded values in the table represent the best values with the least level of error.

CRC classes	ResNet-164		ResNet-152		ResNet-110		ResNet-101		ResNet-50		dResNet-101	
	Min	Max	Min	Max	Min	Max	Min	Max	Min	Max	Min	Max
Class 1	0.013	0.022	0.016	0.026	0.018	0.031	0.024	0.036	0.034	0.052	**0.011**	**0.017**
Class 2	0.013	**0.018**	0.016	0.024	0.021	0.033	0.026	0.038	0.032	0.058	**0.013**	0.019
Class 3	0.012	0.019	0.017	0.023	0.022	0.030	0.028	0.036	0.035	0.049	**0.008**	**0.014**
Class 4	0.011	**0.014**	0.013	0.019	0.018	0.029	0.026	0.035	0.035	0.044	**0.008**	0.014
Class 5	0.014	0.021	0.015	0.023	0.020	0.034	0.028	0.039	0.038	0.056	**0.012**	**0.018**
Class 6	**0.009**	0.018	0.013	0.025	0.022	0.028	0.025	0.038	0.036	0.046	0.010	**0.016**
Class 7	0.010	0.017	0.011	0.021	0.023	0.029	0.030	0.036	0.041	0.051	**0.009**	0.014
Class 8	0.011	0.014	0.016	0.019	0.025	0.033	0.024	0.037	0.035	0.054	**0.007**	0.012
Average	0.011	**0.013**	0.014	0.022	0.021	0.031	0.026	0.037	0.035	0.051	**0.009**	0.014

**Table 5:** In this table, the outcomes of CRC classification utilizing HI for the second dataset with nine classes are presented. The bolded values in the table represent the best values with the least level of error.

CRC classes	ResNet-164		ResNet-152		ResNet-110		ResNet-101		ResNet-50		dResNet-101	
	Min	Max	Min	Max	Min	Max	Min	Max	Min	Max	Min	Max
Class 1	0.001	0.004	0.001	0.005	0.001	0.005	0.003	0.008	0.003	0.010	**0**	**0.002**
Class 2	0.001	0.005	0.001	0.005	0.002	0.006	0.003	0.009	0.004	0.012	**0**	**0.001**
Class 3	**0**	0.003	0.001	0.004	0.001	0.006	0.003	0.010	0.004	0.012	0.001	**0.002**
Class 4	**0**	0.004	0.002	0.005	0.002	0.007	0.005	0.011	0.006	0.013	0.001	**0.003**
Class 5	**0.001**	0.004	0.002	0.006	0.003	0.007	0.006	0.011	0.006	0.014	0.001	**0.002**
Class 6	0.001	0.005	0.002	0.006	0.003	0.007	0.005	0.012	0.007	0.014	**0**	**0.001**
Class 7	**0.003**	0.006	0.003	0.006	0.005	0.008	0.006	0.010	0.007	0.013	0.003	**0.004**
Class 8	0.003	0.006	0.003	0.007	0.004	0.008	0.008	0.009	0.008	0.013	**0.002**	**0.004**
Class 9	0.003	0.006	0.004	0.007	0.004	0.009	0.008	0.012	0.009	0.014	**0.002**	**0.004**
Average	0.0014	0.0047	0.002	0.0056	0.0027	0.007	0.0052	0.0102	0.006	0.0127	**0.0011**	**0.0025**

Several methods have been used in the field of feature extraction by pre-trained models, but the models based on the ResNet structure are not only accurate in creating suitable features, but have shorter processing times due to their light structure. Because ResNet creates separable features, Tables 4 and 5 indicate that the structure of ResNet is the most appropriate model for creating a satisfactory output. It only takes a few minutes to produce meaningful feature maps of a histopathological image using the proposed model and a limited number of replications. Even though deeper structures may in some cases contribute to better features, some transfer-based learning methods have a lot of processing time (especially during training) and may not be suitable for real-time or near-real-time applications.

**Figure 7:**
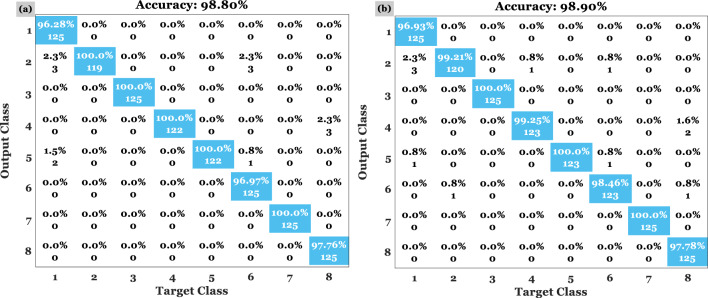
This figure illustrates the confusion matrix resulting from the proposed model's implementation on the first dataset (i.e., with 8 classes) for (**a**) validation and (**b**) test.

**Figure 8:**
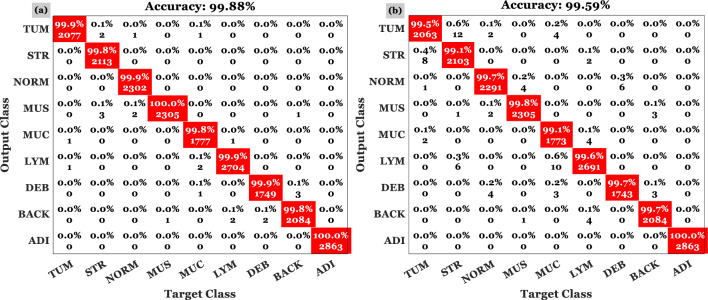
This figure shows the confusion matrix resulting from the proposed model's implementation on dataset NTC-CRC-100 (i.e., with 9 classes) for (**a**) validation and (**b**) test.

In Figs. 7 and 8, confusion matrices for the first and second HI datasets are shown. To determine the decision-making procedure and keep time processing in the final experiment, only the minimum of selected features were employed. Several experiments have demonstrated that 98.82–99.76% of the CRC can be correctly classified in HI. The classification process was performed on two datasets using 50 features based on a specific number of iterations and a variety of CRC related issues. Moreover, both forms were assigned appropriate categories based on the outcomes of the test.

**Figure 9:**
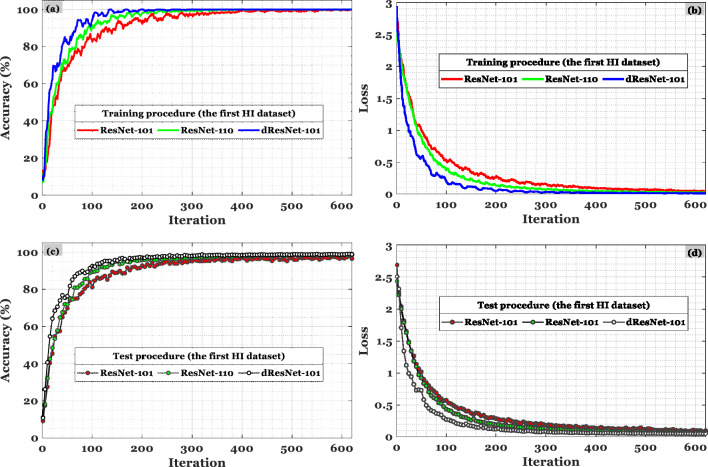
In the CRC-5000 dataset, dResNet-101 was compared with ResNet-101 and ResNet-110 based on the accuracy in (**a**) and (**c**) and the loss in (**b**) and (**d**) for train and test data, respectively.

For the validation data, the average accuracy of 98.80% is shown in the confusion matrix on the left of Fig. 8 in the first scenario. Based on the second scenario (i.e., confusion matrix on the right side of Fig. 7 based on the test data), the accuracy is 98.90%. Likewise, Fig. 8 shows the algorithm being implemented on the second HI dataset. As well, the method is verified based on the unseen HI categories, which is estimated to be 99.89% and 99.67%, respectively, for the test and validation data on the right and left side of Fig. 9. The HIs were correctly classified in 99.76% of the 9 tissue types. Even though the results are less accurate than the first dataset, the addition of automatically extracted features and ensemble learning significantly improves classification accuracy. A second dataset validation using a large number of HIs is used in part 2 in order to demonstrate the same level of confidence in the classification technique. In the first dataset, the proposed strategy is 98.8% accurate while in the second dataset, it is 99.77% accurate.

With the method outlined here, one can identify different types of CRC tissues. Moreover, it was able to obtain a 99% accuracy rate while dealing with a wide range of tissue classification. In particular, the model was 99% accurate in several subtypes of classification of CRC. It is possible that the proposed feature extraction and improved learning algorithms will achieve a satisfactory level of certainty and generalization. Hence, the outcomes demonstrate that the suggested approach is competitive in the detection and classification of colorectal cancer.

The accuracy and precision rates of various approaches are commonly degraded when the HI contains complicated segments and tissues (e. g., when the images contain inappropriate color and improper distribution of illumination). Meanwhile, the method for analyzing HIs is resilient and dependable, and is therefore an efficient and effective method.

### Ethical approval

The use of data is standard and similar to valid research and there is no conflict of interest, ethical or legal issues. Data used in this paper is publicly available and derived from a study by Kather and et al.^40^, whose tests have been approved by the Medical Ethics Board (Medical Ethics Board II, University of Mannheim Medical Center, University of Heidelberg, Germany; 2015 certification -868R-MA). In regards to their data, it is mentioned that the Organizational Ethics Board ignored the need for informed consent in reviewing these anonymous examples retrospectively. In addition, all tests were conducted according to approved instructions and the Helsinki Declaration.

## Discussion

Adopting the proposed approach for detecting and classifying HIs is fundamentally dependent on its ability to differentiate tumors and colon tissues appropriately into two or more classes. Consequently, the expert physician will be able to make a better diagnosis and, on the other hand, continuous monitoring of people will be possible. Listed below are the reasons for employing each method.

Diagnoses depend heavily on past knowledge and are unquestionably essential. Pathologists examine microscopic properties of cells, such as size, shape, texture, colour, and blackness, in order to diagnose diseases. According to previous research, the HSV colour space, which is denoted by the letters H, S, and V in the HSV colour space, can be used to store and transmit colour data without loss. As a result of its spectrum of colours, HIs can distinguish between malignant and healthy cells. A different colour would alter the overall tone of the histopathology image, so maintaining the current one is crucial. For colour representation, HSV is superior to RGB because it accurately depicts how humans see colour. The lack of contrast and inadequate illumination sometimes make it difficult to identify HIs. Due to its out-of-range nature and color fidelity, the HSV colour model was presented.

**Figure 10:**
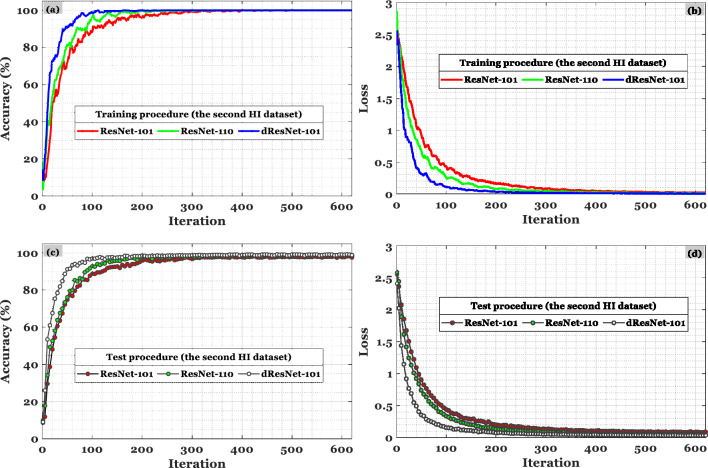
In the NCT-CRC-HE-100K dataset, dResNet-101 was compared with ResNet-101 and ResNet-110 based on the accuracy in (**a**) and (**c**) and the loss in (**b**) and (**d**) for train and test data, respectively.

With the help of dResNet, we can generate features that are both helpful and computationally efficient. Due to ResNet's architecture, it is possible to rapidly construct a network with several layers while lowering training and testing errors. In ResNets, identity mapping is the key to solving the vanishing gradient problem. To counteract disappearing gradients, ResNet-101, ResNet-110, and ResNet-164 use residual blocks. In the revised ResNet-101, several convolutional layers comprise the remaining bottleneck blocks. Convergence speed and consistency of deep learning algorithms vary. Compared with ResNet-101 and ResNet-110, dResNet-101 produced more informative features and achieved convergence more rapidly. The recommended CRC classification method had to be stable and reliable as the number of HIs classifications increased in order to provide the most accurate results. A deep learning model's accuracy and loss estimates indicate that the first group of HIs examined in the early portion of Fig. 9 tend to be the most accurate. The accuracy of classification in the other two sections of Fig. 10 is also acceptable when trained on accuracy and loss computations.

**Figure 11:**
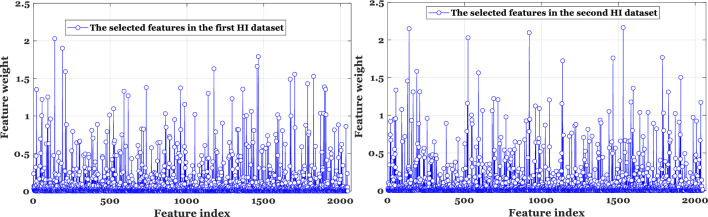
It is evident from this figure that a variety of features were selected by the NCA, but high classification accuracy was still achieved with as few features as possible.

Overall, it was found that feature selection did not have a significant impact on classification accuracy. The primary reason for this is that dResNet only extracts features that distinguish. Figure 11 shows that the NCA technique selected a variety of features, yet high classification accuracy was achieved with a minimal number of features.

In addition to being more effective, some of the features can also be more efficient. NCA has been shown to be an extremely powerful way of selecting the optimal subset of features, as well as a nonparametric method of selecting features to maximize prediction accuracy.

**Figure 12:**
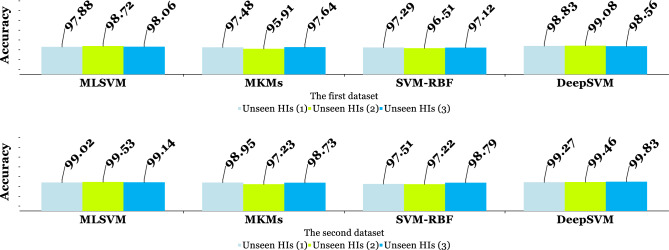
In both datasets, DeepSVM classification was compared to other similar methods for classifying three unseen HIs.

DeepSVM's classification ability was demonstrated in numerous experiments by Qi et al.^46^. Based on evaluations of various datasets, they compared the DeepSVM method to similar approaches, such as Multi-layer SVM (MLSVM)^49^, Multi-layer kernel machines (MKMs)^50^, and SVM with RBF kernel. An experiment was also conducted using three unseen collections of histopathology images in order to compare these approaches to the proposed method. For comparison, these methods were chosen since they are capable of classification and, on the other hand, are highly comparable to DeepSVM. Figure 12 compares the performance of classification using a limited set of features for both datasets as unseen histopathology images.

Contrary to Eed-to-End (e2e) and Softmax structures, this process reduces computing complexity while maintaining competent processing speed and accuracy. Another experiment evaluated the performance of the suggested approach in terms of feature extraction as well as the final classification while also addressing the issue of computational complexity. According to Table 6, several transfer learning algorithms based on extractive features have different computational complexity and accuracy. Model engineering (ME) architectures were used to estimate the runtimes of numerous transfer learning models.

**Table 6:** The following table compares how accurate the extracted features are and how computationally complex they are. Computing times are an average of three training runs using various TL models.

TL network	Dataset	Accuracy	Runtime-training	Runtime-test (s)	Computational complexity
Inception v4	Kather texture 2016	97.31	20 min, 58 s	0.36	Low
	NCT-CRC-HE-100K	98.47	7 h, 16 min, 29 s	0.38	
VGG-16	Kather texture 2016	97.93	23 min, 44 s	0.33	Medium
	NCT-CRC-HE-100K	98.86	8 h, 09 min, 27 s	0.34	
VGG-19	Kather texture 2016	98.08	27 min, 51 s	0.46	High
	NCT-CRC-HE-100K	99.17	9 h, 33 min, 12 s	0.43	
DenseNet-169	Kather texture 2016	98.48	50 min, 38 s	0.76	High
	NCT-CRC-HE-100K	99.57	19 h, 40 min, 30 s	0.72	
DenseNet-201	Kather texture 2016	98.54	1 h min, 16 s	1.12	High
	NCT-CRC-HE-100K	99.68	22 h, 06 min, 13 s	1.43	
ResNet-101	Kather texture 2016	98.30	49 min, 26 s	0.61	Low
	NCT-CRC-HE-100K	99.29	18 h, 36 min, 44 s	0.64	
ResNet-110	Kather texture 2016	98.43	54 min, 12 s	0.67	Low
	NCT-CRC-HE-100K	99.35	20 h, 54 min, 21 s	0.72	
ResNet-152	Kather texture 2016	98.64	1 h, 17 min, 28 s	0.75	Medium
	NCT-CRC-HE-100K	99.42	23 h, 35 min, 37 s	0.78	
ResNet-164	Kather texture 2016	98.73	1 h, 32 min, 20 s	0.83	High
	NCT-CRC-HE-100K	99.63	26 h, 03 min, 41 s	0.89	
dResNet-101	Kather texture 2016	98.80	1 h, 07 min, 04 s	0.66	Medium
	NCT-CRC-HE-100K	99.79	21 h, 23 min, 31 s	0.69	

As well, we examine our method in light of the most recent research on CRC classification based on deep learning. Table 7 shows the statistical assessments made in NCT-CRC-HE-100K. Chen et al.^37^ and Ghosh et al.^51^ developed an approaches that has resulted in better results in recent years. Compared to Chen et al.^37^, our strategy outperforms another based on average accuracy.

**Table 7:** Based on the NCT-CRC-HE-100 K and Kather texture 2016 images, a comparison is made of quantitative methods and our proposed model.

Method	Dataset	Model	Accuracy (%)	Generalizability	Computational complexity
Ghosh et al.^51^	NCT-CRC-HE-100K	Ensemble learning based on the CNN	96.16	Medium	High
Hamida et al.^52^	Kather texture 2016	ResNet and TL	96.60	Medium	High
	NCT-CRC-HE-100K	ResNet and TL	99.76	Medium	High
Kather et al.^53^	NCT-CRC-HE-100K	VGG-16 and TL	98.70	Medium	Low
Chen et al.^37^	NCT-CRC-HE-100K	MCAM	99.68	Medium	High
		IL-MCAM	99.78	Medium	High
Alqudah et al.^41^	Kaggle	QDA	97.30	Medium	Low
Kumar et al.^43^	NCT-CRC-HE-100K	NCT-CRC-HE-100K	99.21	Medium	High
The proposed model	Kather texture 2016	dResNet and DeepSVM	98.75	High	Low
	NCT-CRC-HE-100K		99.76	High	Low

According to Chen et al.^37^, the suggested architecture has an average accuracy of 99.78%, which is 0.02% lower than IL-MCAM and 0.06% higher than MCAM. Our method is more generalizable than their models, and the computational complexity of the proposed method has been reduced substantially. Additionally, they did not mention asymmetrical light distributions and color intensifications caused by staining, as well as the excessive tissue complexity of the histopathological image.

As the early diagnosis of colon tumors is crucial, precise and fast classification of HIs is a critical step in cancer detection. Accurate classification algorithms must perform effectively in the absence of annotated datasets to minimize pathologists' workload. The hybrid strategy demonstrated in this study was suggested as a generalizable procedure for overcoming time and learning constraints, as well as to facilitate the deployment of HI images in clinics and for accurate classifications.

## Conclusion

We introduced a hybrid architecture that incorporates dilated DTL processes and effective learning to classify colorectal cancer (CRC) based on histopathological images. The suggested framework uses a dilated ResNet-101 model that incorporates an attention module for automatic feature generation and learning to analyze colorectal cancer histology texture. A NCA-based feature selection procedure was utilized to reduce the computational complexity after the features were extracted. The selected feature was then fed to a robust DeepSVM classifier to classify colorectal cancer multi-class texture. The suggested method outperforms traditional deep learning models in both training and testing. Consequently, the improved model is a reasonable solution for identifying and classifying colorectal cancer. In contrast to other similar methods, which cannot be generalized and are subject to uncertainty, the proposed method often does not compromise accuracy when complex sections and textures are included in HIs. In extensive experiments using Kather_texture_2016 and NCT-CRC-HE-100K datasets, the generalizability of the proposed framework is demonstrated. As a comparison, we used many unseen collections of histology images in the experiment. In the future, we will combine and permute attention mechanisms with deep learning models to select the optimal model. Further, we will investigate the effects of convolutional layers on classification performance by incorporating attention mechanisms into separate aspects of deep learning models.

## Data Availability

This dataset was taken from University Medical Center Mannheim (Germany)^47^, accessible from "https://zenodo.org/record/53169#.Y8pHeXZBzIU", and NCT-CRC-HE-100K^48^, accessible from "https://zenodo.org/record/1214456#.Y8pHc3ZBzIU". Codes are also available from the corresponding authors.
